# Planetary extravehicular activity (EVA) risk mitigation strategies for long-duration space missions

**DOI:** 10.1038/s41526-021-00144-w

**Published:** 2021-05-12

**Authors:** Blaze Belobrajdic, Kate Melone, Ana Diaz-Artiles

**Affiliations:** Department of Aerospace Engineering, Texas A&M University, College Station, TX United States

**Keywords:** Aerospace engineering, Physiology, Risk factors

## Abstract

Extravehicular activity (EVA) is one of the most dangerous activities of human space exploration. To ensure astronaut safety and mission success, it is imperative to identify and mitigate the inherent risks and challenges associated with EVAs. As we continue to explore beyond low earth orbit and embark on missions back to the Moon and onward to Mars, it becomes critical to reassess EVA risks in the context of a planetary surface, rather than in microgravity. This review addresses the primary risks associated with EVAs and identifies strategies that could be implemented to mitigate those risks during planetary surface exploration. Recent findings within the context of spacesuit design, Concept of Operations (CONOPS), and lessons learned from analog research sites are summarized, and how their application could pave the way for future long-duration space missions is discussed. In this context, we divided EVA risk mitigation strategies into two main categories: (1) spacesuit design and (2) CONOPS. Spacesuit design considerations include hypercapnia prevention, thermal regulation and humidity control, nutrition, hydration, waste management, health and fitness, decompression sickness, radiation shielding, and dust mitigation. Operational strategies discussed include astronaut fatigue and psychological stressors, communication delays, and the use of augmented reality/virtual reality technologies. Although there have been significant advances in EVA performance, further research and development are still warranted to enable safer and more efficient surface exploration activities in the upcoming future.

## Introduction

Humans have an innate desire to explore, but this aspiration to understand the unknown is not without its risks. Space exploration is one of the most challenging and dangerous endeavors to embark on; therefore, reducing risks of planetary extravehicular activities (EVAs) is crucial in order to enable such exploration. In order to mitigate these risks, it is important to define and categorize the known risks. In doing so, astronauts will be equipped with both the necessary technology and skills to overcome obstacles that will inevitably arise on long-duration space missions.

Spacesuits have unique challenges owing to the extreme environments in which they are used. The extravehicular mobility unit (EMU) was designed for low earth orbit (LEO), and has been NASA’s operational spacesuit since 1983. As humans travel back to the Moon and eventually to Mars, future spacesuits will encounter additional obstacles during EVA in planetary environments. Considering only a few surface EVAs occurred over the entirety of the Apollo program, the anticipated increase in EVA quantity will require robust spacesuits capable of long-endurance planetary mission scenarios.

Spacesuit design encompasses both material selection of the spacesuit, which is important to consider for radiation shielding and dust mitigation, as well as all the internal systems that support the regulation and monitoring of physiological health such as hypercapnia prevention, thermal control, and others. In addition, spacesuits must be functional for the astronaut wearing it and the design must take into account the environment(s) in which the astronaut will be operating. In addition to spacesuit design, the concept of operations (CONOPS) is an integral part of successful EVAs. Considerations that affect CONOPS include the actual EVA in terms of duration and difficulty of tasks, as well as other areas that impact EVA performance, such as fatigue and psychological well-being. Although international space station (ISS) EVAs are an extremely strenuous task for astronauts, planetary EVAs may present an even greater challenge. Future exploration EVA missions, such as Artemis, will require more mobility than current ISS requirements, as mobility at higher gravity levels, such as on the surface of Mars, will require the wearer to move with more gravitational load across sloped terrain with varying surface properties. This in turn will impact the metabolic workload of the crew member and will impact both the design of the spacesuit as well as mission CONOPS^1^.

The goal of this research effort is to identify the key risks associated with planetary EVAs and to identify suitable mitigation strategies, specifically for planetary surface exploration. Through an extensive review of ongoing research that includes academia, government, and industry, this paper will pinpoint some of the key risks associated with EVAs, with emphasis on those for planetary surface exploration. In this context, we divided EVA risk mitigation strategies into two main categories: (1) spacesuit design and (2) CONOPS. Table 1 indicates the specific considerations associated with each category that are further developed in the rest of this document.

**Table 1:** Summary of research considerations and associated research items.

Mitigation strategies	Research consideration	Research items
Spacesuit design	Hypercapnia prevention	Support liquid membranes, swing bed scrubber, mask sensor system
	Thermal regulation and humidity control	Spacesuit water membrane evaporator, full-body radiator, liquid cooling ventilation garment, variable geometry radiators
	Nutrition, hydration, and waste management	Maximum absorbency garments, wastewater stabilization
	Health and fitness requirements	High-intensity interval training, emergency procedures for incapacitated crew
	Decompression sickness	Exercise pre-breathe protocol, hypobaric environment
	Radiation shielding	Radiation Protection Garment (PERSEO Project), biological countermeasures, magnetic shields, hydrogenated boron nitride nanotubes, FLARE Suit
	Dust mitigation strategies	Spacesuit integrated carbon nanotube dust ejection/removal, electrodynamic dust shield, photovoltaic dust removal technology, electron beam
	Health monitoring and injury prevention	Biosensor, bioharness, astroskin, lifeguard, warfighter physiological status monitoring, glucowizzard, “Lab-on-Skin” Devices, BioSuit
Concept of operations	Astronaut fatigue	Schedule logistics, task assignments, suit mass reduction
	Psychological well-being	Assessment of autonomy, competence, and relatedness
	Operational challenges	Heads-up display, augmented reality, holo-sextant, communication methods

## Spacesuit design

Spacesuits are vital for EVA as they serve as the astronaut’s own personal spacecraft. How well a suit is designed can facilitate or hinder the success of an EVA. This section focuses on some of the greatest risks associated with EVAs, specifically related to crew health. Strategies to address some of the potential problems that could arise are also presented in this section.

### Hypercapnia prevention

The spacesuit circulation system is intended to prevent the buildup of waste gases, namely CO_2_, that are hazardous to health in large concentrations. Hypercapnia can occur during EVA by re-inhaling local concentrations of CO_2_ left by inadequate airflow or failure of the suit to scrub excess CO_2_^2^. The EMU’s portable life support system (PLSS) removes CO_2_ and excess water vapor while providing thermal protection. The next-generation exploration PLSS (xPLSS) includes the swing bed scrubber (SBS) along with the ventilation test loop 2.0 (VTL2) to remove both CO_2_ and water vapor from the ventilation loop^3^. The SBS was built for the Constellation Space Suit System (CSSS) but did not undergo performance testing. Supported liquid membranes (SLMs) can remove ammonia (NH_3_) and formaldehyde (CH_2_O) which, according to recent studies, can exceed their spacecraft maximum allowable concentrations if not handled properly^4,5^. The trace contaminants inside the ISS have a separate filtration system not suited for EVA, and so the PLSS (and eventually the xPLSS) must filter harmful trace contaminants. Researchers are utilizing ionic liquid sorbents to reduce the average permeance value of CH_2_O and NH_3_ without significant loss of O_2_ in the process^5^.

Measurement of CO_2_ buildup carries considerable variability based upon component composition (such as sample line length or placement of flow controllers)^6^. A standardized CO_2_ washout measuring method reduced sampling-induced errors and should assist future spacesuit development and set exposure standards^7^. In addition, the CO_2_ washout measurement was further refined by the substitution of a nasal cannula; however, this was done in a controlled experimental environment, and may not be suitable for EVAs with higher energy expenditure levels^8^. Nose-only breathers demonstrate greater CO_2_ washout owing to increased tidal volume, decreased respiratory rate, and exhalations that are directed away from the nasal region. A recent study presented a streamlined method for determining partial pressure-inspired CO_2_ (${\mathrm{P}}_{{\mathrm{ICO}}_2}$) to ensure safe levels for existing and future spacesuits^9^. Another relevant technology being developed is the pilot mask sensor (MASES) system, which provides on-board, real-time monitoring of pilot breathing gas. The mask has an embedded probe with luminescence sensors to measure relative humidity, pressure, temperature, pCO_2_, and pO_2_. Real-time monitoring of pCO_2_ and pO_2_ allows closed-loop control of the on-board O_2_ generation system and is based upon the pilot’s respiration^10^. Although the mask was developed for military aviators, the system has application to EVA. As spacesuit design continues, the inclusion of systems capable of monitoring the inlet and outlet gases, while providing data on metabolic expenditures, can increase planetary EVA safety.

### Thermal regulation and humidity control

Spacesuit thermal regulation systems maintain internal temperature for astronaut safety and comfort. The internal equilibrium balances the variations in the metabolic heat expenditure with environmental sink temperatures external to the suit. At present, this is accomplished through the use of a liquid cooling and ventilation garment (LCVG), which regulates crew member temperature by running chilled water via tubes with skin contact. The LCVG and PLSS have reliably provided the primary means of thermal regulation for the EMU and Russian Orlan spacesuits, but will require mass reductions and redesign to accommodate EVA on the surface of Mars. It has been observed that performance decrements manifest above 480 Btu/hour heat storage and tissue damage begins at 800 Btu’s heat storage^11^. During the Apollo lunar surface EVAs, heat expenditure rates ranged from 780 to 1200 Btu/hour^11^. As EVA duration and task requirements increase, so will the rate and amount of total heat expended. It will become imperative to understand and quantify estimated heat expenditure values prior to planetary EVAs to ensure that the crew can maintain proper body temperature, nutrition, and hydration standards.

To address concerns for thermal regulation, researchers from MIT conducted a thermal management technology review coupled with a Technology Readiness Level (TRL) assessment of those technologies^12^. The review included solid-state and phase-change heat exchangers, variable geometry radiators, variably emissive electrochromic radiator devices, and evaporative cooling. The evaporator technology is furthest along in development with the spacesuit water membrane evaporator (SWME) having flown on an ISS payload flight test in 2019^13^. However, none of the technologies have met the current heat rejection goal of 250–300 W for an 8-hour EVA^14^. An alternative suit approach may be a mechanical counterpressure (MCP) suit. An MCP suit can be thought of as a “second skin”, as it requires a skin-tight fit that entails a detailed understanding of human skin deformation, especially at the joints, to ensure anthropometric parity and unmitigated locomotion. The suit works by compressing the body, rather than pressurizing a spacesuit garment^15^. Some of the key advantages of the MCP suit over a traditional gas-pressurized suit are increased joint mobility and decreased total suit weight/bulk^15^. Because the astronaut would not be enclosed in a pressurized suit environment, the MCP suit would allow for more “natural cooling.” As the astronaut sweats, the water droplets would pass through the MCP suit and evaporate into the atmosphere, therefore dissipating heat and cooling the astronaut^15^. UC Boulder and the Technical University of Munich have evaluated the capabilities of spacesuit cooling via a full-body radiator concept that considers both the gas pressure and MCP suits in Lunar and Martian environments^16^. The MCP suit presented performance fluctuations with higher metabolic rates, whereas the gas pressure suit presented fluctuations with wind speed. However, the MCP suit has some drawbacks including difficulties with uniform compression and suit donning/doffing^15^. The MCP suit also has unanswered questions regarding technology integration, and therefore this area warrants further investigation and future work.

UC Boulder has also investigated thermoelectric devices for temperature control. Their method works by altering thermal loading using variable emissivity films, which through modulation of emissivity, can change how much heat is being retained or lost^14^. This could aid in regulating body temperature during various metabolically taxing activities (such as EVAs) and could accommodate external temperature changes in the environment without the need for an LCVG. The time it takes for such a concept has been demonstrated in the order of seconds, and while this device has not been approved for spaceflight yet, it has been evaluated in space-like environments and shows promise for future missions^14^. Another approach involves the use of variable infrared emissivity electrochromic materials (pixels) to actively modulate heat rejection^17^. This constant temperature architecture rejected 100–500 W using an emissivity range of ~0.169–0.495^17^. The constant heat flux variation allowed for the same amount of metabolic workload rejection (100–500 W), but used a variable emissivity range of 0.122–0.967^17^. Both of these approaches have no loss of consumables, less overall mass, and no system power requirements^17^.

An alternate approach to thermal regulation and humidity control involves the reimagination of the LCVG through the inclusion of water-permeable membranes that assist with water vapor absorption^18^. The multifunctional cooling garment is meant to prevent condensation buildup inside the garment, utilize regenerable CO_2_ removal beds to prevent water loss, and conserve water through a lithium chloride absorber/radiator technology^18^. Another study evaluated wearer-controlled vaporization, via the self-perspiration for evaporative cooling garment, (or SPEC-W), and compared results from a baseline study (no cooling), simulated LCVG, and SPEC-W^19^. It was shown that the SPEC-W alone was effective in lowering skin temperature^19^. Another added benefit of the SPEC-W over the LCVG is that cooled the wearer without increasing the humidity inside the garment (which could be a contributing source to discomfort while wearing an LCVG for an extended period of time)^19^. Further investigation of alternate cooling methods for EVAs could lead to more efficient systems and increased suit comfort, which will become more important as the duration and frequency of EVAs increase.

### Nutrition, hydration, and waste management

The removal of spacesuit waste in a safe and efficient manner is a very critical part of EVAs. Shorter EVA durations partly solve the need for waste management systems, but as operational requirements dictate longer EVAs, this will not be an option. To manage in-suit elimination, maximum absorbency garments (MAGs) are worn during EVA and used, if necessary^20^. MAGs have the side effect of causing discomfort, skin irritation, and an unpleasant odor^21^. If astronauts are required to remain in their spacesuits owing to an emergency, the need for efficient waste management systems could become life-critical, as skin exposure to feces and urine leads to compromised skin integrity, leaving the dermis open to infection^22^. One technique that astronauts use to avoid this problem is to reduce their consumables intake through fasting before an EVA to correspondingly reduce the likelihood of elimination during EVA^23^. There is no evidence suggesting that task performance suffers as a result of the astronauts’ fasted state in microgravity environments. However, task performance may suffer in higher-g environments as the metabolic workload will most likely increase from microgravity. For example, a 10-km walk-back test, simulating a lunar surface on-foot trip to a lunar module from a broke-down rover, demonstrated an increased requirement for nutrition and hydration^24^. Average calories burned were 944 kcal and all subjects felt additional food and drink would improve endurance and performance, indicating that fasting prior to EVA may not be a sufficient mitigation strategy. The environmental control and life support technology gaps are being addressed at the macro level for intravehicular activity (IVA) and include multi-filtration bed and urine processor assembly upgrades, brine dewatering development, biological water processing, and wastewater stabilization^25^. Although the technologies are not meant for EVA, they will support EVA operations and should eventually scale to spacesuit development, addressing the waste management concern (and in turn removing the need for fasting prior to EVA). The development and implementation of these technologies will play an important role in future planetary surface explorations and will enable safer, longer-duration EVAs.

### Health and fitness requirements

Long-term space missions on the ISS have demonstrated deteriorating function on the muscle-tendon unit structure, decrease in bone density, poor sleep quality, cardiovascular deconditioning, central nervous system changes, and neuro-ophthalmic changes^26–28^. To address the relationship between crew member health and fitness with operations in exploratory environments, the Crew Health and Performance EVA Systems Maturation Team (CHP EVA SMT) seeks to better understand the deleterious effects of spaceflight. The CHP EVA SMT is also involved in the development of new tasks and procedures for debilitated crew member operations. Crew health and fitness will also be important when the crew first arrives at their destination. For example, when astronauts return to Earth from the ISS, there are medical professionals to assist with crew egress. On Mars, the crew will not have that luxury. Even though Martian surface gravity is ~0.376 of Earth’s so the transition from microgravity to Mars might not be as drastic as when the crew returns to Earth from LEO, maintaining physical health during transit will still be vital. The implementation of current (e.g., exercise, nutrition) and future (e.g., centrifugation^29–31^) countermeasures is an important factor to consider. High-intensity interval training combined with artificial gravity may help mitigate cardiovascular deconditioning and promote aerobic fitness, both of which will be especially vital for physically intense planetary EVA^29,32,33^.

### Decompression sickness

Spacesuits can be considered single-person spacecrafts that provide an atmospheric environment to perform required tasks. Proper spacesuit pressure is critical to avoid body fluid vaporization (>0.9 psia) and prevent decompression sickness (DCS)^4^. DCS results when nitrogen bubbles form in the tissue. The nitrogen bubble buildup in the bloodstream leads to rashes, tissue damage, joint pain, and degraded neurological function^34^. Maintaining the same pressure between the spacecraft (or habitat) and spacesuit negates the nitrogen buildup, but it is not practical in terms of mobility/pressurization tradeoffs, as high pressurization can induce fatigue and an increased probability of suit rupture^1,35,36^. Although NASA has a relatively low DCS prevalence rate with no astronauts having experienced DCS in space and few in training^34^, it is still a significant risk owing to the severity of the consequences if DCS did occur in space. In addition, a joint medical research team identified the possible increase in decompression illness as humans travel beyond LEO, so it is will become even more important to manage on future long-duration missions^37^.

DCS mitigation has long been and will continue to be a concern for preserving astronaut health. Future research tasks include the development and evaluation of DCS risk models and validated procedures to prevent DCS. Two common exercise pre-breathe protocols are the “cycle ergometer with vibration stabilization” and “in-suit light exercise”, which have both been used extensively on ISS^35^. However, current DCS mitigation strategies used in microgravity will not be suitable for planetary surface exploration, and will need to be modified for safe and logistically feasible EVAs^38,39^. New EVA protocols for an exploration atmosphere (8.2 psia at 34% O_2_) may be required to reduce DCS risk, as well as mild hypoxia, spaceflight associated neuro-ocular syndrome, and acute mountain sickness^40,41^. Researchers at the High Altitude Pulmonary and Pathology Institute in La Paz, Bolivia have investigated a different exploration atmosphere, and instead, make the case for a hypobaric environment of 9.5 psi and 20.9% O_2_^42^. In addition, it will also be important to consider time requirements for EVA preparation. The frequency of EVAs performed will increase^23^, and spending hours per day to engage in pre-breathe activities will not be logistically realistic. Further research will be critical in developing safe suit conditions and efficient operation logistics for planetary EVAs.

### Radiation shielding

The risk of radiation exposure in both deep space and on a planetary surface is primarily mission-dependent. Space radiation includes solar particle events (SPEs) and galactic cosmic radiation (GCR), and consequences of exposure fall into four major categories: (1) carcinogenesis, (2) degenerative tissue risk, (3) acute and late risks to the central nervous system, and (4) acute radiation syndrome (ARS)^43^. Highly energetic heavy ions, or HZE charged particles, are also hazardous to astronauts and their equipment, justifying the need to protect crew members and their critical electronic components^44^. Researchers at NASA have also reviewed current ARS biomathematical models and recommended the utilization of on-board dosimeter input for estimating both radiation doses to organs and the most probable outcomes^45,46^. Planetary EVAs should be planned around solar activity, but not all SPEs and GCRs are predictable, and so carcinogenesis risk mitigation is necessary for lunar visit/habitation, deep space journey/habitation, and planetary missions^47^.

The EMU’s material layup includes a Thermal Micrometeoroid Garment (orthofabric composed of Teflon/Nomex/Kevlar, Reinforced Aluminized Mylar, and neoprene-coated nylon ripstop), Dacron polyester, urethane-coated nylon ripstop, and the LCVG. The Orlan-DM, however, incorporates several layers of polyethylene for radiation dose reduction^48^. Polyethylene merges a high level of hydrogenation, is affordable, machines well, and is the material effectiveness standard^49^. Kevlar, which provides shielding from debris, also exhibits radiation shielding properties^50^. The Personal Radiation Shielding for interplanetary missions (PERSEO) project, led by the Italian Space Agency, developed a simulated radiation protection garment filled with water to shield crew member’s organs during SPEs^51^. The water can then be recycled to optimize the use of available resources^51,52^. The simulated prototype reduced radiation dose levels by 40% to blood-forming organs and the gastrointestinal tract. Although the system was designed for IVA, the concept can scale to EVA. Another IVA concept transferable to EVA is the FLARE Suit, proposed by a researcher at the KTH Royal Institute of Technology. It consists of multiple bladders that can quickly be filled with saltwater, which act as shielding against neutrons^53^. Another potential radiation countermeasure may be the inclusion of hydrogenated Boron Nitride Nanotubes (BNNT) in either the surface lander module, or the spacesuit itself. Compared with carbon (which serves as one of the base elements in polyethylene), both boron and nitrogen have greater neutron absorptions capabilities, with boron having one of the largest out of all the elements. Initial testing of BNNT within the context of long-duration spaceflight applications has been conducted at MIT^54^. Results from this study indicate that hydrogen-enriched BNNT is comparable to polyethylene in terms of SPE shielding effectiveness (90.0% and 90.1%, respectively), but shows more of an improvement with respect to GCR radiation shielding (23.2% for hydrogenated BNNT, 16.7% for polyethylene)^54^. Radiation shielding alone may not be enough, thus alternative radiation protection methods could also be necessary. These include biological countermeasures against radiation such as antioxidants (such as Vitamin C), Neulasta (a bone marrow stimulant), topically applied steroid creams, antibiotics, and tardigrade DNA (injected into tissue). Active radiation shielding, such as electrostatic and magnetic shielding, should also be researched for possible countermeasures against radiation damage^55^. Electrostatic shielding operates on the principle of creating a lens of gossamer membrane structures to deflect incoming GCR via multiple charged spheres in certain orientations^55^. Magnetic shielding through either a superconducting solenoid around the spacecraft or mini-magnetospheres (and toroidal magnetic fields) deployed further from the spacecraft could deflect radiation below certain thresholds^56^. Both may be applicable to protecting crew members during EVA operations and reduce the likelihood of radiation damage. However, these concepts are both at a low TRL and extensive research and development are still required in order for these concepts to be implemented on a spacecraft.

### Dust mitigation strategies

Lunar dust resulted in deleterious effects on Apollo-era spacesuits such as fabric abrasion, clogging seals, and the potential to restrict visibility^57^. During surface EVA, dust particles are transported into the spacecraft and/or habitat. Specifically, lunar dust caused irritation to unsuited astronaut eyes and sinuses^58^ and led some Apollo-era astronauts to remain suited in the lunar module, after witnessing the accumulated dust floating in the cabin, to prevent dust inhalation and entering their eyes/sinuses. Dust interaction can be characterized by an abrasion index (including abrasion mode severity, particle interaction frequency, hardness of mineralogy, and risk level) and by zone (either outside the spacecraft, in transitional areas, or inside the habitat)^59^. Abrasion can be evaluated in both two- and three-body interactions to quantify volumetric material wear and to identify which materials are best suited for the lunar and Mars environments^60^. Dust abrasiveness and granularity also have crew health implications (like respiratory illness) inside the habitat^61^. In fact, the greatest risk to the lung is a combination of altered pulmonary deposition (owing to physiological changes induced by microgravity) with planetary dust and the possibility for that dust to be highly toxic^62^. Perchlorates in the Martian dust are a concern and must be sufficiently removed from EVA suits prior to habitat re-entry in order to prevent inhalation of the harmful particles and contamination of the habitat^63^. A sufficient mitigation strategy for removing perchlorate is simply washing it off, as the perchlorates would dissolve in the water^63^. Establishment of an acceptable baseline particle load, as well as and mitigation procedures and equipment, is fundamental for future planetary EVA exploration on the Moon.

Researchers at the University of Southern California discovered that there is a serious risk at the lunar terminator (i.e., dividing line between day and night) from electrostatic discharge^64^. One lunar anti-dust technology is the Spacesuit Integrated Carbon Nanotube Dust Ejection/Removal (SPIcDER) system, which is designed to protect spacesuit outer surfaces^65^. The system uses an electrodynamic dust shield (EDS) and work function matching coating concepts, developed at NASA, that repel lunar dust via carbon nanotube yarn^66^. An alternative to the SPIcDER system is a new photovoltaic lunar dust removal technology, utilizing a dust-removing electrode (composed of the photoelectric material lanthanum-modified lead zirconate titanate) that polarizes for dust removal^67^. Researchers in China optimized a comb-shaped electrode by varying material parameters, dust-removing electrode area, comb-tooth width, and gap width found that constant area, 1 mm tooth width, and 1 mm gap width resulted in near 100% dust removal efficiency improvement^67^. A second study was conducted varying particle size and charge and found that a 320 mm × 125 mm dust-covered surface could achieve 95% dust mitigation^68^. Another method of dust mitigation is to remove the particles by causing them to “jump off” the surface via an electron beam^69^. This approach would specifically target dust <25 μm in diameter, as particles of this size have typically been more challenging to remove with previous dust removal strategies^69^. Results show that the electron beam approach removed ~75–85% of the dust particles over the course of ~100 seconds^69^. With an increased frequency in planetary surface operations, effective dust mitigation strategies will be vital to protect both the crew and the integrity of the spacesuit.

### Health monitoring and injury prevention

EVAs are extremely strenuous, and can lead to fatigue, decrease in performance, and astronaut injury^70,71^. One of the most commonly reported EVA injuries (both for training and actual ISS operations) are hand trauma injuries^72–75^. Most of the current EVA operations heavily involve astronauts to use their hands, both for manipulating tools, but also for traversing along the ISS. During planetary EVAs, additional injuries may become more prevalent as other health risks are likely to be introduced owing to surface gravitational effects. Crew health monitoring within the spacesuit could provide invaluable information about astronaut and spacesuit health and thus, it could prevent and protect against EVA-related injuries. NASA aims to create an internal suit sensor suite to characterize human performance during EVA and provide crew member biometric tracking, which includes a radiation-hardened biosensor^76,77^. Sophisticated spacesuit sensor suites will need to provide enough data to assist researchers and provide crew member surveillance tracking while not adversely affecting spacesuit costs, mass and power reserves, and crew member performance. Several health monitoring systems, most of which were originally created for military and aviation personnel, can monitor stress and vital signs within the spacesuit environment (BioHarness, Astroskin, LifeGuard, and Warfighter Physiological Status Monitoring)^78–81^. Vital sign measurements include: heart and respiration rate, body motion and position, fluid intake, skin temperature, and sleep estimates via actigraphy. An implantable biosensor such as the Glucowizzard, could be placed to monitor specific biomarkers like blood glucose levels, and provide constant data without the need to consider skin-to-skin contact of a biosensor^80^. In addition, “Lab-On-Skin” devices exhibit physical properties similar to the human epidermis and are able to measure physical parameters, such as temperature and blood pressure^82^. In addition to these physiological measurements, a proximity electromagnetic resonant spiral sensor has been developed to monitor the relative spatial position of an astronaut’s shoulder to the scye bearing joint^83^. The purpose of this sensor is to reduce the likelihood of astronaut shoulder injuries (including skin abrasions or more serious rotator cuff tears), which primarily occur in the presence of gravity, such as during donning/doffing of the spacesuit during training on Earth, and potentially during future planetary surface exploration where the crew will be subjected to the weight of the suit^83^.

Another potential injury prevention strategy could be the inclusion of an MCP system. The BioSuit is an MCP spacesuit concept that could be used to enhance suit mobility and thus human performance during EVA^84^. The MCP concept is still in the early stages of development and has a low TRL. However, hybrid spacesuit concepts (combining both gas pressure and MCP) are promising, since they feature higher mobility employing more reasonable levels of MCP^1,36^.

Quantifying spacesuit mobility is also important as reduced range of motion can lead to injury. One study used accelerometers and gyroscopes (which utilized inertial measurement units and relative rotation) on both the inside and outside of a MK-III suit, and results showed differences between the spacesuit and the human joint angles. They also identified statistically significant impairment of mobility between the pressurized and baseline conditions^70,85–87^. Robotic actuation may also assist with mobility and with injury prevention. One study conducted at Texas A&M University using OpenSim software to model the EMU indicated that the inclusion of robotic actuation in lower extremities during planetary ambulation could reduce metabolic cost by ~15%, which could lead to increased efficiency and less chance of an astronaut over-exerting himself/herself during EVAs^1,88^.

## EVA operational risks

Another critical aspect to ensuring crew safety and mission success during planetary EVAs is the CONOPS of the mission, as well as understanding and accounting for environmental risks. Analog sites on Earth, such as Hawai’i Space Exploration Analog and Simulation (HI-SEAS), McMurdo Station, NASA Extreme Environment Mission Operations (NEEMO), and Human Exploration Research Analog (HERA) help to understand some of the psychosocial effects of being in an isolated and confined environment for an extended period of time. This section focuses on some of the lessons learned from analog missions on Earth.

### Astronaut fatigue

Some of the greatest risks associated with planetary EVAs include astronaut fatigue and injury, which could potentially lead to incapacitation or inability to return to the surface habitat. Astronaut fatigue can occur from a variety of sources, including but not limited to poor sleep, long working hours, mental fatigue from performing monotonous routine tasks or exceptionally difficult tasks, and physical fatigue from physically strenuous EVAs. Some of these risks can be avoided through careful design of an astronaut’s schedule, but others like physical exhaustion might not be directly avoided through scheduling. Future long-duration missions to the Moon or Mars will require astronauts to perform up to 24 h of EVA per week, which is significantly greater than the typical three to four EVAs astronauts perform during a 6-month ISS stay^23^. Although the duration of an EVA cannot be relied upon as a way to reduce risk, EVA performance logistics and methods can be adjusted.

One of the main factors that contribute to astronaut fatigue is increased metabolic workload during EVAs. This increase in workload can come from suit weight, suit pressure, and suit mobility, with the largest factor being suit weight^23^. There are two primary ways of reducing metabolic workload: (1) employ walking speed requirements or (2) reduce suit mass. It has been shown that there exists a direct correlation between metabolic workload and speed, regardless of if the subject is suited or unsuited^23^. If, for example, during an EVA, astronauts are not required to adopt a faster than normal pace (i.e., normal conditions, no emergency), reduced walking speed will suffice in mitigating the risk, making suit mass/planetary suit weight not as important of a consideration. However, in an emergency scenario, astronauts may be required to return to the habitat as quickly as possible, and thus, solely relying on walking speed as a countermeasure against increased metabolic workload would not be sufficient. Therefore, total suit mass reduction to decrease metabolic workload, thus reducing the risk of compromised astronaut health during EVAs, should be considered. Researchers at the Space Systems Laboratory at the University of Maryland, College Park investigated an innovative concept called BioBot^89^. In this spacesuit architecture, the life support equipment is primarily carried by the rover instead of the astronaut, therefore reducing PLSS mass^89^. Ten potential astronaut life support system configurations were considered, ranging from a minimal mass configuration of approximately 18 kg that could supply 20 min of life support operated in an open loop, to a maximum mass configuration of ~68 kg that could provide up to 480 min of life support operated in a closed loop^89^. Depending on the type and duration of the EVA, the astronaut wears a custom PLSS configuration, in addition to being tethered to BioBot^89^. The multiple configurations allow for the astronaut to not be subjected to unnecessary weight while traversing the surface^89^.

### Psychological well-being

Understanding psychological needs become especially important for long-duration spaceflight, especially for a mission to Mars, as the crew will not have access to most of the current ISS mitigation strategies (i.e., real-time conversations with family, viewing Earth, etc.). One of the mission objectives of HI-SEAS IV was to evaluate the self-determination theory in order to better understand how astronauts will react to the presence, or lack of thereof, of certain psychological needs^90^. The three areas of focus of the study were autonomy, competency, and relatedness^90^. The study found that the crew members who were more satisfied in the autonomy, competency, and relatedness tended to perform better individually, but also with others^90^. In addition, they felt less stressed and were deemed less likely to rebel against instructions they received^90^. These results are important for EVA risk mitigation because the success of an EVA is highly dependent on the crew. The importance of relatedness and the positive group dynamic was shown to have a direct impact on a crew member’s performance, which ties into EVA safety and success^90^.

### Operational challenges

Workload reduction systems that decrease cognitive loading and maximize available cognitive capacity will mitigate EVA risks associated with EVA operations, including mission duration and safety gaps. EVA navigation in unknown planetary terrain will be dangerous, tedious, and task saturating. An investigation on multimodal displays found that the use of vibrotactile feedback in reduced visibility environments for obstacle cues and avoidance minimized heads-down time, created a more conservative gait, and optimized foot placement^91^. Another team of researchers proposed the concept of haptic feedback (or a periphery cap system) as a means of relaying information to the crew member^92^. The cap would have a haptic language of its own and would assist in the filtration of sensory input through the layers of the spacesuit^92^. In addition, the haptic cap may reduce the cognitive load for the user since information normally transferred through touch will now have a channel to the user inside the suit^92^. Future spacesuit design incorporating a heads-up display (HUD) and augmented reality (AR) may demonstrate utility for space exploration. HUD’s have demonstrated workload reduction and orientation assistance in aviation for decades^93^. Texas A&M University researchers are investigating the use of VR and AR technologies for space exploration^94,95^. One particular study investigated the use of AR to display information using a Microsoft Hololens, and results showed that AR has promise in reducing task completion time, which could improve the overall efficiency of EVA^94^. Scaling HUDs for use in spacesuits will allow crew members excess capacity for cognitive loading and increased situational awareness.

Another proposed system, the Holo-Sextant (Surface Exploration Traverse Analysis and Navigation Tool), was tested under the BASALT project in 2017 using HoloLens technology^96^. The Holo-Sextant uses a graphic terrain overlay and can calculate the shortest route to traverse. The tool displays waypoint features, distance traveled, distance to/from set locations, time measurements, and interfaces with a mobile application. The Holo-Sextant system also decreased user cognitive workload, easing stress, and allowing focus on other tasks. The visual interface was of minimalist design and could provide checklist procedures, basic navigation, communications, and spacesuit information.

An additional operational challenge will be communication. Although Lunar missions will still essentially have real-time communication with Mission Control on Earth, a Mars mission will be subject to time delays and a higher degree of autonomy^97,98^. These time delays could not only lead to frustration, but also misinterpretation of tasks and overall reduced EVA efficiencies^99^. Studies conducted under the BASALT project specifically investigated how communication would affect the CONOPS of a Mars planetary EVA through the implementation of two one-way light time delays of 5 min and 15 min^99,100^. BASALT investigated several modes of communication, including traditional voice communication, as well as GPS tracking, still imagery, video transmission, and text messages^99^. One of the major findings from the study was the importance of text-based communication. Unlike all previous space missions, which primarily relied on voice communication, text messages were found to be the most useful. Not only do they provide written documentation in a Mission Log for future use, but the crew was able to prioritize tasks, rather than sticking to a set schedule^99^. This could allow for more crew autonomy and independence from mission support teams, which could benefit crew morale, and also potentially increase EVA efficiency^99^. GPS tracking methods may also be beneficial for navigation and orientation assistance. Although the crew would not receive real-time support, if Mission Control noticed the crew deviating from the intended path, or was approaching a potentially hazardous area, a message could be sent, and a plan could be implemented in order to return to the desired course. GPS tracking could be used in conjunction with some of the other navigation/orientation assistance methods discussed and could be used as a redundant system in case one of the other methods failed.

## Conclusion

Some of the key findings from recent and current research in academic institutions, government, and industry regarding spacesuit design and mission CONOPS for successful planetary EVAs have been summarized. Mitigation strategies within the context of spacesuit design and the operational context were also presented. The combination of new technologies related to spacesuit design and new operational considerations, including improved scheduling logistics and task assignments, will better equip astronauts for the incredible challenges associated with planetary EVAs. Further research on these (and other) mitigation strategies is still warranted to enable safer and more efficient surface exploration activities in the future.

## Data Availability

Data sharing not applicable to this article as no data sets or codes were generated as a result of this research effort.
